# Early CT and MRI signs of invasive fungal sinusitis complicating COVID-19 infection: case report

**DOI:** 10.1186/s43163-022-00206-0

**Published:** 2022-01-29

**Authors:** Ahmed Samir, Mohamed Said Abdel-Gawad, Amr Magdy Elabd, Walid Mohamed Abed, Ayman Mahmoud, Tamer Yousry Gaweesh, Ahmed Youssef

**Affiliations:** 1 Department of Radio-diagnosis, Faculty of Medicine, Alexandria University, Alexandria, Egypt; 2 Department of Radio-diagnosis and Intervention, National Liver Institute, University of Menoufia, Shebeen El-Kom, Egypt; 3 Department of Radio-diagnosis, Medical Research Institute, Alexandria University, Alexandria, Egypt; 4 Department of Otolaryngology & Head and Neck Surgery, Faculty of Medicine, Alexandria University, Alexandria, Egypt

**Keywords:** COVID-19, Early signs, Invasive fungal sinusitis, Mucormycosis, Case report

## Abstract

**Background:**

Corticosteroids are usually prescribed for severe cases with SARS-CoV-2 (COVID-19). Despite their importance to decrease patients’ mortality, they can cause serious side effects like fulminant fungal infection that can damage lungs or invade the sinuses then rapidly spread to the orbit and even intra-cranially. Unless early diagnosed and properly managed, patients can lose their vision or die from cavernous sinus thrombosis or other intracranial complications.

**Case presentation:**

A 71-year-old diabetic male patient presented with dry cough, fever, and dyspnea for 6 days. PCR test for COVID-19 was ordered and declared positive. The oxygen saturation on day 7 started to decline to reach 90%. Eight ampules of intra-muscular dexamethasone were prescribed. The patient’s dyspnea improved, and the oxygen saturation reached 94% by day 13. Oral prednisone was prescribed in a withdrawal protocol. Unfortunately, on day 15, the patient complained of mild left-sided cheek swelling and noticeably dropped left angle of mouth. Neurological consultation suspected facial palsy and asked for brain MRI examination. Limited lower cuts of the MRI study that covered the left maxillary antrum revealed mild fullness of the pre-maxillary fat planes with mucosal thickening. Complimentary dedicated MRI and CT cuts over the left maxillary sinus showed localized signs of invasive fungal sinusitis without orbital or intracranial complications. The patient received antifungal therapy even before evident endoscopic findings appeared. He underwent endoscopic debridement few days after and he had an excellent outcome without any progression or significant morbidities.

**Conclusion:**

Early CT/MRI radiological signs of invasive fungal sinusitis that complicated COVID-19 infection aid in the diagnosis and proper timely management of this fatal disease.

## Background

Corticosteroids are usually prescribed for severe cases with SARS-CoV-2 (COVID-19) who developed rapid oxygen desaturation [1]. Despite their importance to decrease patients’ mortality, they can cause harmful side effects, especially in immunocompromised patients. Additional risk factors include uncontrolled diabetes mellitus and neutropenia with or without malignant diseases [2]. One of these side effects is serious, and even fatal is fulminant fungal infection that can damage the lungs or invade the maxillary sinuses then rapidly spread to the orbital cavities and even intra-cranially [3, 4]. Unless early diagnosed and properly managed; patients can lose their vision or die from pulmonary hemorrhage or intracranial complications [5]. Also, radical surgical interventions including radical maxillectomy and orbital exenteration for the sino-orbital involvement would be mandatory with questionable outcomes and frustrating facial dysmorphism [6].

The previous literature demonstrated the late cases of sino-orbital mucormycosis. The authors in this case report differently provided the CT and MRI diagnostic signs for early isolated sinus involvement. The authors aimed to highlight the importance of the early clinical and radiological diagnosis before the development of the notorious CT or MRI signs of bony destruction, orbital or intracranial invasion. This early diagnosis could eventually change the clinical decisions and hilariously modify the patients’ outcomes.

## Case presentation

**Fig. 1:**
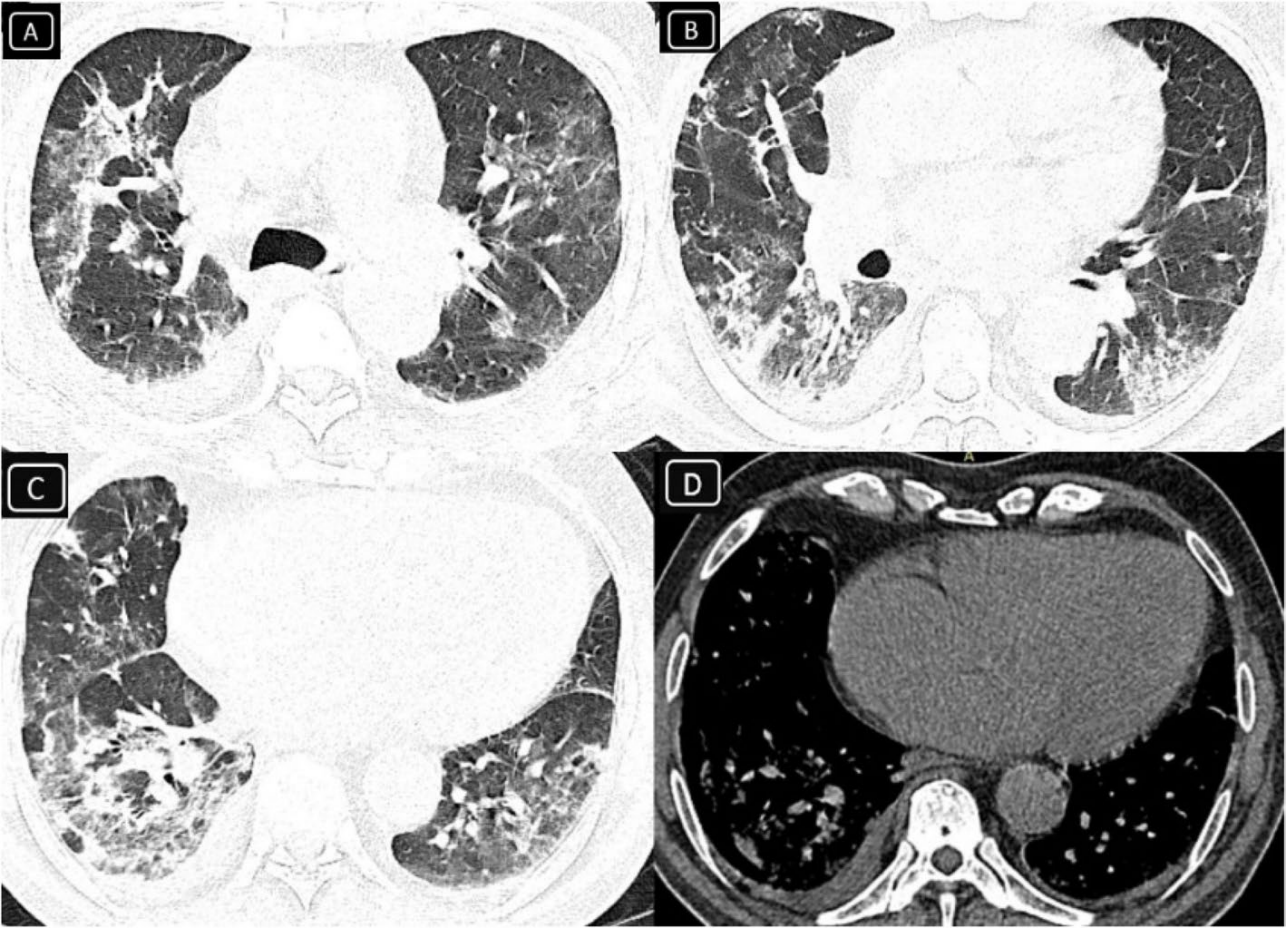
**A**–**C** Axial non-contrast chest CT (lung window) revealed bilateral widespread peripheral ground glass patches with septal thickening and peri-lobular fibrosis with architectural distortion (classic CT signs of severe COVID-19 infection). **D** Axial non-contrast chest CT (mediastinal window) also revealed mild right-sided and minimal left-sided pleural collection with cardiomegaly

**Fig. 2:**
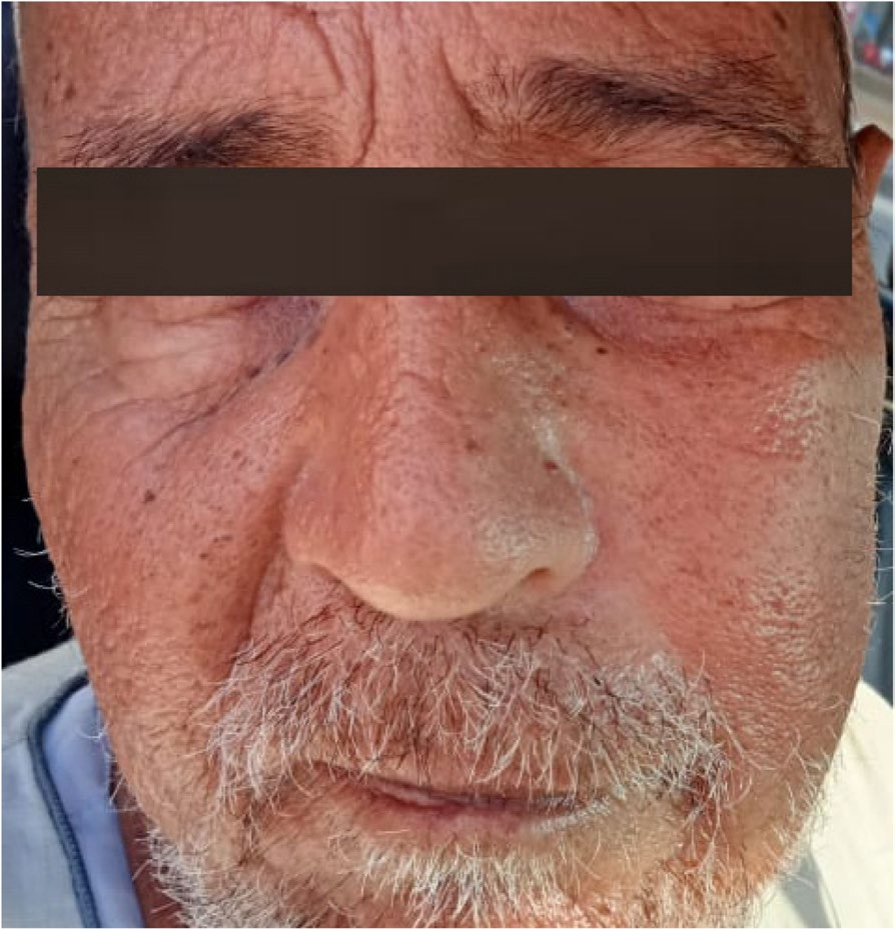
Mild asymmetric left cheek swelling was the only early clinical complaint

**Fig. 3:**
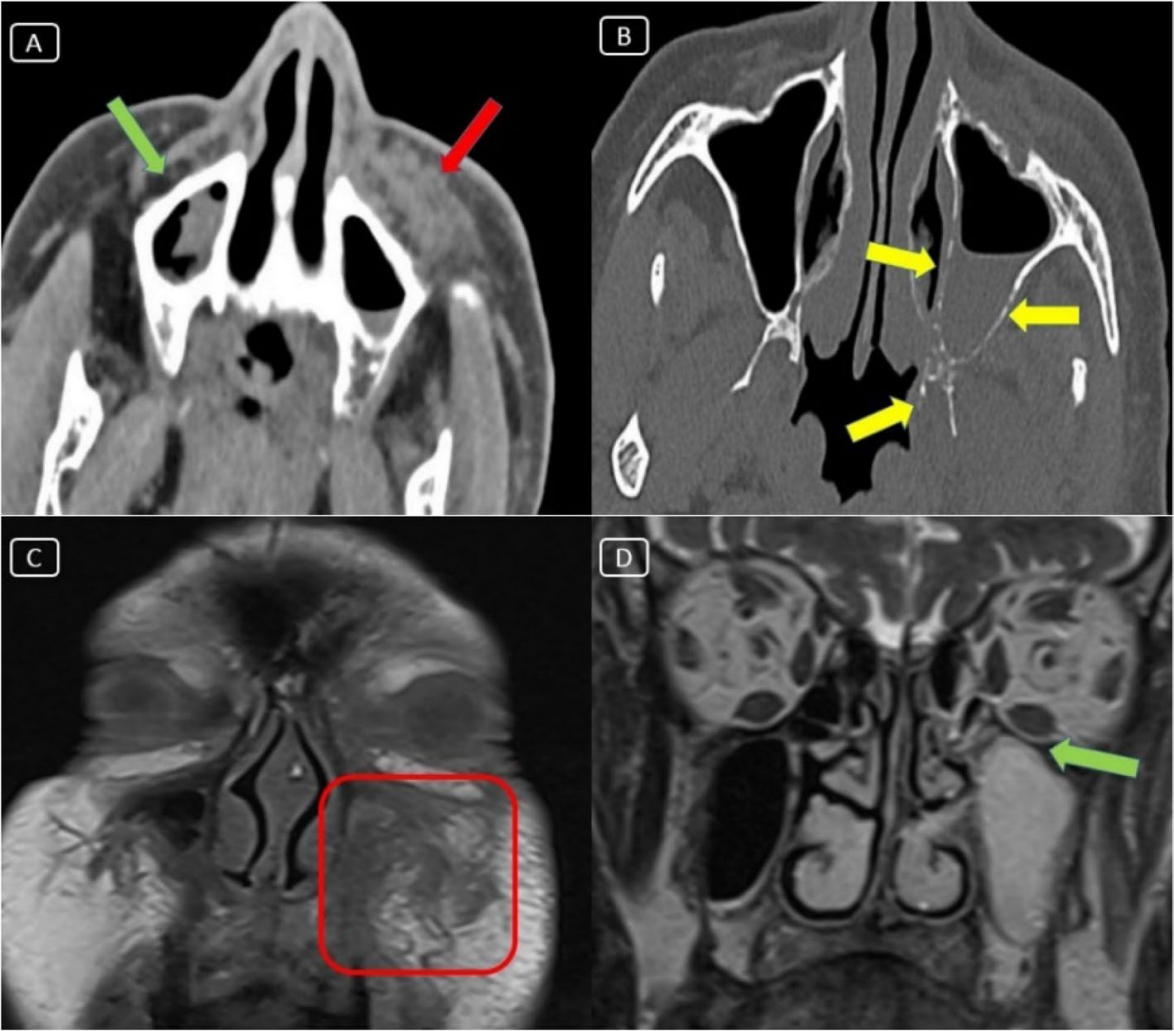
**A**, **B** Axial CT cuts of the paranasal sinuses and orbital regions revealed (1) soft tissue fullness replacing the left pre-maxillary antral fat (red arrow), (2) mild antral mucosal thickening, and (3) thinning out of the medial and lateral maxillary bony walls with areas of dehiscence and rarefaction that extended posteriorly up to the root of the pterygoid plates (yellow arrows). **C** Coronal T1WI MRI of the paranasal sinuses revealed asymmetrical fullness and heterogeneous signal intensity of the premaxillary fat (red square). **D** Coronal T2WI MRI of the paranasal sinuses and orbital regions revealed left maxillary sinusitis still with clear peri-orbital fat planes (green arrow)

A 71-year-old diabetic male patient presented with dry cough, fever, and dyspnea for 6 days. His CBC laboratory examination revealed leukopenia (WBC = 2.8 × 10^−3^/μl) without lymphopenia (lymphocytes 58%). COVID-19 infection was suspected, and a PCR test for COVID-19 was ordered and declared positive. Home isolation and symptomatic treatment started immediately. The oxygen saturation on day 7 started to decline to reach 90%. Non-contrast chest CT examination was performed. It showed classic CT features of severe COVID-19 infection (Fig. 1). These CT findings included the following: (1) bilateral widespread peripheral ground glass patches with septal thickening and peri-lobular fibrosis with architectural distortion, (2) reactive mediastinal nodes, (3) mild right-sided and minimal left-sided pleural collection, and (4) cardiomegaly. Eight ampules of intra-muscular dexamethasone were prescribed at home when the patient refused hospital admission. Unfortunately, the patient neglected to monitor his blood sugar levels. The patient’s dyspnea lately improved, and the oxygen saturation reached 94% by day 13. Oral prednisone was then started in a withdrawal protocol. Unfortunately, on day 15, the patient complained of mild left-sided cheek swelling (Fig. 2) and noticeably dropped left angle of mouth without ocular symptoms or signs. The initial neurological consultation suspected facial palsy and asked for a brain MRI examination. Lower cuts of this study that cover the left maxillary antrum revealed mild fullness of the pre-maxillary fat planes. Complimentary dedicated MRI and CT cuts over the left maxillary sinus showed localized signs of invasive fungal sinusitis without orbital or intracranial complications (Fig. 3). The MRI findings included the following: (1) left maxillary sinusitis was noted with air-fluid leveling and mucosal thickening that compromised the ostio-meatal complex, (2) fullness and heterogeneous signal of the premaxillary fat with relative mild veiling of the retro-antral fat planes, (3) the left orbital fat planes were still clear, and (4) mild synchronous right maxillary antral mucosal thickening still with clear pre- and retro-antral fat planes. The CT confirmed the MRI findings and added the presence of thinning out of the medial and lateral maxillary walls with small focal areas of bony dehiscence and mild rarefaction that extended posteriorly to the root of the pterygoid plates as well as the left orbital floor. Consultation of ENT service for endoscopic evaluation was achieved. There was no evidence of black eschar along turbinate or nasal septum; however, with high index of suspicion, amphotericin B was immediately prescribed for our patient side by side to insulin injections to control blood sugar levels. Few days after, endoscopic surveillance showed black eschar over middle turbinate and middle meatus on the left side, so the patient was generally fit for an urgent limited endoscopic debridement. The patient did very well for the next weeks after, and he did not need any further procedures.

## Discussion

Since declaring the COVID-19 pandemic; respiratory complications were the most common cause of human fatalities [7]. Corticosteroids played a very important role in the modulation of the immune-mediated lung damage caused by the COVID-19 virus and hence decreased human mortality [8]. On the other hand, the incidence of superadded fungal infection had increased especially in the immuno-compromised patients [8, 9]. It could affect the lung yielding a fatal process of alveolar hemorrhage. It also could involve the upper respiratory tract and paranasal sinuses. The invasive form of sinusitis “mucormycosis” caused by this fungal infection can destruct their boundaries with the further spread of infection to the orbital cavities and intra-cranially either directly or along valve-less veins in head and neck region [10]. The patients can lose their vision because of direct infection, optic nerve involvement, or cavernous sinus thrombosis [11]. Furthermore, orbital exenteration could be mandatory during the process of necrotic tissue eradication, which results into a great morbidity to these patients [12]. Additionally, the mortality rates significantly rise as a result of intracranial involvement [13]. Early diagnosis and management can cause a dramatic decrease in morbidity and mortality rates including reversal of immune-suppression states, early antifungal therapy, and surgical debridement of necrotic and fungal tissues [14]. Furthermore, early diagnosis can limit the extent of surgical treatment to endoscopic debridement and avoid major procedures that often result in disfigurement and function loss.

Most of the previously published case reports or case series about mucormycosis and COVID-19 infection exhibited the late and complicated forms of the disease with pulmonary, sino-orbital, or intracranial involvement. CT picture of pulmonary alveolar hemorrhage was described with dense ground-glass patches. MRI signs of the sino-orbital invasion included orbital cellulitis, inflammatory pseudo-tumor, optic hydrops, and exophthalmos. MRI signs of enhancing meningo-encephalitic patches were also demonstrated. Radical maxillectomy with orbital exenteration was the main surgical line of treatment [5, 15–21].

In this case report, the authors provided the early CT and MRI signs for isolated sinus infection. These radiological signs even were present few days before the appearance of endoscopic signs of invasive fungal sinusitis. Eventually, early diagnosis leaded to timely proper clinical management in this case which saved the patient from the intracranial complications and frustrating outcomes such as vision loss and facial dysmorphism.

## Conclusions

Early CT/MRI radiological signs of invasive fungal sinusitis that complicated COVID-19 infection like fullness and heterogeneous signal of the premaxillary fat with relative mild veiling of the retro-antral fat planes in addition to thinning out of the maxillary sinus walls with small focal areas of bony dehiscence aid in diagnosis and proper timely management of this fatal disease.

## Data Availability

The data and material are available from the corresponding author on reasonable request.

## Abbreviations

PCR: Polymerase chain reaction
ENT: Ear, nose, and throat
